# Applications of Infrared and Raman Spectroscopies to Probiotic Investigation

**DOI:** 10.3390/foods4030283

**Published:** 2015-07-17

**Authors:** Mauricio I. Santos, Esteban Gerbino, Elizabeth Tymczyszyn, Andrea Gomez-Zavaglia

**Affiliations:** 1 Center for Research and Development in Food Cryotechnology (CIDCA, CCT-CONICET La Plata), 1900 La Plata, Argentina; E-Mails: maurysantos@hotmail.com (M.I.S.); estebanq2001@hotmail.com (E.G.); 2 Laboratory for Molecular Microbiology, Department of Food Science and Technology, National University of Quilmes, 1876 Buenos Aires, Argentina; E-Mail: elitym@yahoo.com.ar

**Keywords:** FTIR (Fourier Transform Infrared Spectroscopy), NIR (near infrared spectroscopy), Raman spectroscopy, probiotics, multivariate analysis

## Abstract

In this review, we overview the most important contributions of vibrational spectroscopy based techniques in the study of probiotics and lactic acid bacteria. First, we briefly introduce the fundamentals of these techniques, together with the main multivariate analytical tools used for spectral interpretation. Then, four main groups of applications are reported: (a) bacterial taxonomy (Subsection 4.1); (b) bacterial preservation (Subsection 4.2); (c) monitoring processes involving lactic acid bacteria and probiotics (Subsection 4.3); (d) imaging-based applications (Subsection 4.4). A final conclusion, underlying the potentialities of these techniques, is presented.

## 1. Introduction

Probiotics are “live microorganisms that, when administered in adequate amounts, confer a health benefit on the host” [1]. Most of probiotic strains belong to the genera *Lactobacillus* and *Bifidobacterium*. Among the beneficial effects of probiotic strains, one can mention immunological stimulation, improvement of digestion and absorption, synthesis of vitamins, inhibition of potential pathogens, decrease of cholesterol levels, lowering of gas distension, and restoration of the normal flora after antibiotic therapy [2]. Certain species of the genera *Bifidobacterium* and *Lactobacillus* are common inhabitants of the intestinal microbiota. Its development is modulated and controlled by complex mechanisms including intrinsic biological regulatory functions, genetic endowment and environmental constraints [3,4]. In addition to these factors, the intake of probiotics through the diet and/or through pharmaceutical products plays a critical role for the development of intestinal microbiota.

These physiological functions outline the importance of probiotic strains as food ingredients and as pharmaceutical supplements, thus supporting their production at an industrial level [3]. In addition, the increasing consumer awareness about gut health has strongly contributed to the increase of probiotics’ demand.

The global market of probiotics is continuously growing and is expected to attain 33.6 billon Euros in 2018. The total demand of probiotics for the food and beverages segment is estimated to reach 28.4 billion Euros in 2018. Probiotics are widely used in dairy products, cereals, baked products, fermented meat products, dry foods and other less relevant products. Dairy products are the largest market for probiotic foods, their demand being estimated to reach 24.1 billion Euros in 2018. Probiotics have also emerged as a critical part of the animal feed industry. In this latter case, the demand is estimated to be above 2.2 billion Euros by 2018 [5,6].

This enormous importance of probiotics market underlines the need of reliable methods to evaluate their properties both at a research and at an industrial level. Infrared and Raman spectroscopies were originally used almost exclusively in fundamental Physics and Chemistry. However, the development of strong equipment together with appropriate computational resources for the analysis (occurring mainly in the last 20 years) led to a continuous broadening of applications in biological and biomedical fields. In this review, we will focus on the use of infrared and Raman spectroscopies for the study of probiotics and lactic acid bacteria.

## 2. Brief Overview of the Fundamentals of Infrared and Raman Spectroscopies

Conventional methods used to analyze lactic acid bacteria and probiotics include plate counts, molecular biology, and immunology based techniques [7]. Although these methods are very useful and reliable, they are invasive and time-consuming, and thus not suitable for the increasing demands of real time and on-line analyses of large amounts of samples.

The development of strong equipment has definitely contributed to fulfill these demands. Infrared and Raman spectroscopies have several advantages over other methodologies, namely ease-of-use, minimal or no preparation of samples, quick registration of spectra *in situ* and in real time without using chemical reagents (environmentally friendly methodologies) [8]. Furthermore, almost any sample (*i.e.*, liquids, solutions, pastes, powders, films, fibers, gases and surfaces) can be investigated by carefully selecting appropriate sampling techniques.

Using infrared and Raman spectroscopies for the investigation of biological systems, including lactic acid bacteria and probiotics, is an increasingly growing approach. In fact, taxonomic studies, monitoring probiotics production, evaluation of biochemical and biophysical bacterial properties are some of the main applications.

The interaction of matter with light in the infrared region of the electromagnetic spectrum gives rise to the experimental spectra. Infrared and Raman spectroscopies are part of the so-called vibrational spectroscopic techniques. In both of them, measurements correspond to the infrared region of the electromagnetic spectrum. The infrared region can be divided in: near (750–2500 nm), mid (2500–40,000 nm), and far-infrared (40,000–60,000 nm) [9]. This classification is important for the development of equipment.

The spectrum represents the transmitted, reflected or dispersed radiation as a function of energy or a magnitude proportional to it (wavenumber, frequency, wavelength, energy). Infrared features originate from changes in the electric dipole moments during vibrations. In other words, the interactions of infrared radiation with matter may be understood in terms of changes in molecular dipoles associated with vibrations and rotations. In turn, Raman spectra originate from an inelastic scattering of the incident light. Hence, the intensity of the scattered light as a function of the Raman shift is plotted in the spectra. Similar to the infrared spectra, Raman provides information based on stretching and bending vibrational modes as a function of the frequency or other related magnitude.

In spite of these similarities, the information provided by Raman and infrared spectra is complementary rather than identical. In fact, infrared absorptions require changes in the intrinsic dipole moment with molecular vibration, whereas Raman scattering depends on changes in the polarizability of functional groups when atoms vibrate. This explains why polar groups (*i.e.*, C=O, N-H, O-H) occur as strong infrared stretching bands and non-polar groups (*i.e.*, C=C, C-C, S-S) are intense bands in Raman.

Water deserves special mention because due to its polarity, it strongly absorbs in the infrared spectrum. Therefore, when samples with high contents of water are to be investigated, certain strategies shall be adopted (*i.e.*, use of short path length sample cells or attenuated total reflection accessories), in conjunction with careful subtraction of the water baseline spectrum. On the contrary, water has weak Raman scattering properties and thus, produces less interference in Raman spectroscopy. For this reason, Raman spectroscopy is usually more suitable for the *in vivo* or *in situ* study of biological systems, including foods.

Another possibility to overcome the problem of water is the use of near infrared spectroscopy (NIR). Spectral features in the NIR arise from combinations and overtones of the fundamental vibrations involving C-H, O-H and N-H bonds [10]. Therefore, it is a useful tool to investigate sugars and other polyhydroxilated compounds (*i.e.*, polysaccharides), alcohols, compounds having long hydrocarbon chains (*i.e.*, fatty acids) in a relatively “clean” region, without the interference of other spectral bands occurring in the mid infrared region. Due to the low absorptivity of water in the near infrared region, NIR spectroscopy has been extensively used for the study of aqueous systems. Moreover, the development of fiber optic probes coupled with electronic and mathematical novel developments allows monitoring on line, which is particularly valuable for quality control in food industries [9].

When spatial resolution is desired, novel adjustments are currently available. In particular hyperspectral imaging integrates imaging and spectroscopy, that is, obtaining structural information from the images observed at microscopic level [7]. To this aim, this technique allows the creation of the so-called “hypercube” or “datacube” (x, y and λ) described as one-dimensional spectrum (λ) at every 2D pixel (x, y) [11,12]. Extracting the information contained in the “hypercube” generates spectral images (also known as imaging spectroscopy), enabling the mapping of chemical components in a sample. This technique has been used for several analytical applications, including food, agriculture and pharmaceutical analysis (*i.e.*, determination of chemical attributes, such as lipids, proteins and moisture in red meats, compositional analysis in dairy products, embryo development evaluation in eggs, internal quality assessment in fruits and vegetables, infection detection in poultry and cereals) [7,13,14,15,16,17,18,19,20]. The application of hyperspectral imaging in food microbiology is more recent and its application for the study of lactic acid bacteria and probiotics is still very scarce [7,14].

Table 1 summarizes the occurrence of the main infrared features occurring in biological samples. This information supports the interpretation of spectra of lactic acid bacteria and probiotics. It is important to point out that the environment of samples determines different shifts in the occurrence of certain bands (*i.e.*, bands arising from groups involved in hydrogen bonds). This background is crucial to understand the meaning of physical and biochemical changes occurring in the bacterial environment (*i.e.*, humidity, growth conditions, production of metabolites, *etc*.).

**Table 1:** Assignment of the main bands generally found in Fourier Transform Infrared Spectroscopy (FTIR) spectra of biological samples ^a^.

Wavenumber (cm^−1^)	Assignment
~3500	νO-H
~3200	Amide A of proteins
2959	ν(C-H_3_)as
2934	ν(C-H_2_)as
2921	ν(C-H_2_)as (fatty acids)
2898	νC-H (triple bond)
2872	ν(C-H_3_)s
1741–1715	ν(C-H_2_)s (fatty acids)
~1695	νC=O (carbonic and nucleic acids)
~1685, ~1675	Amide I from antiparallel β-sheets and β-turns of proteins
~1655	Amide I of α-helices of proteins
~1637	Amide I of β-sheets of proteins
1548	Amide II of proteins
1515	“Tyrosine” band
1468	δ(C-H_2_)
~1400	ν(C-O)s of COO^−^
1310–1240	Amide III of proteins
1250–1220	ν(P=O)as of PO_2_^−^
1200–900	C-O-C, C-O dominated by ring vibrations of carbohydrates C-O-P, P-O-P
1085	ν(P=O)s of PO_2_^−^
720	C-H rocking of >CH_2_
900–600	“Fingerprint region”

^a^ ν: stretching; δ: bending; s: symmetric; as: asymmetric. Amide A, I, II and III are typical bands of proteins. Amide A corresponds to νN-H; Amide I, to νC=O of amide groups; Amide II, to νC-N + δN-H coupled out of face, and Amide III, to νC-N + δN-H coupled in face.

## 3. Analysis of the Spectral Information

From a chemical point of view, lactic acid bacteria and probiotics are highly complex systems that originate convoluted infrared and Raman spectra. Therefore, their analysis requires certain pre-treatments and using statistical based methods for the interpretation (chemometrics). Spectral pre-treatments are addressed to minimize the variability derived from methodological conditions. The most used spectral pre-treatments are baseline correction, normalization (to minimize differences originated in the amount of sample used for spectra registration), smoothing (to reduce noise in the signal), and derivatization (first or second derivative spectra), to improve resolution and minimize baseline variability. Only after that, chemometric methods can be safely used. The description of all multivariate based methods is out of the scope of this review. In this section, an overview of methods most widely used for the study of lactic acid bacteria and probiotics is provided.

Depending on the prior knowledge of elements and relationships, two general approaches can be followed: unsupervised and supervised multivariate analysis [21]. The first group of multivariate methods aims to describe relationships among spectra without requiring previous knowledge of samples. Hierarchical Cluster Analysis (HCA), Factor Analysis (FA) or Principal Component Analysis (PCA) belong to this group.

HCA joints unknown samples into groups known as “clusters”, which indicate mathematical distances or similarity coefficients of data. FA aims to reduce the dimensionality of a data set and detect hidden relationships between variables. A set of correlated variables is thus transformed into a set of uncorrelated hidden variables, known as “factors”, which are ranked in descending order according to their variability. PCA uses orthogonal transformation to convert the spectra (multivariate data) into linearly uncorrelated variables, named principal components (PC). This transformation is defined in such a way that the first PC has the largest possible variance and each subsequent component has, in turn, the highest variance possible under the constraint that it shall be orthogonal to the preceding components. This has the advantage of eliminating multicollinearity when using the PCA results in an analysis of dependence (*i.e.*, regression analysis) [22]. This way, PCA enables grouping samples according to their spectral similarities.

Supervised multivariate methods require a prior knowledge of samples and include Linear Discriminant Analysis (LDA), Canonical Variate Analysis (CVA), Soft Independent Modeling of Class Analogy (SIMCA) or Artificial Neural Networks (ANN). All these methods require a great amount of spectra to define a function or model aiming the identification or classification of unknown samples.

LDA, also known as Fisher Discriminant Analysis, is used for pattern recognition and aims at finding linear combinations of features enabling the characterization or separation of two or more classes of objects. The resulting combination may be used as a linear classifier or to reduce dimensionality before future classifications [23].

CVA aims at determining the relationships between groups of variables in a data set. The data set is splitted into two groups (*i.e.*, X and Y), based on certain common characteristics. The purpose of CVA is to find the relationship between X and Y. It works by finding the linear combination of X (*i.e.*, X_1_, X_2_, *etc*.) and Y variables (*i.e.*, Y_1_, Y_2_, *etc*.), that is most highly correlated. This combination is known as the “first canonical variates”, usually denoted as U_1_ and V_1_, the pair of U_1_ and V_1_ being called “canonical function”. The subsequent canonical functions, U_2_ and V_2_ are then restricted so that they are uncorrelated with U_1_ and V_1_. Everything is scaled, thus, the variance is equal to 1 [23,24].

In SIMCA, a PCA is performed on each class in the data set, and a sufficient number of PCs are retained to account for most of the variation within each class. Hence, a PC model is used to represent each class in the data set. The number of PCs retained for each class is usually different. By comparing the residual variance of an unknown to the average residual variance of those samples that make up the class, it is possible to obtain a direct measure of the similarity of the unknown to the class. This comparison, is also a measure of the goodness fit of a given sample, to a particular PC model [23].

In ANN analysis, the class assignment of each individual object is necessary from the beginning. It is used to estimate functions that may depend on a large number of inputs that are generally unknown. In general, the obtained networks are presented as systems of interconnected “neurons” that send messages to each other. The connections have numeric weights that can be tuned based on experience, making neural nets adaptive to inputs and capable of learning [25].

For a quantitative analysis, a variety of chemometric algorithms, including Multiple Linear Least-Squares (MLR), Partial Seast-Squares (PLS), principle component regression (PCR) analyses, are used to define spectroscopic models [26,27]. In MLR, linear relationships between the absorption in discrete wavelengths of the spectra and information provided by reference analytical chemistry methods are defined. In PLS, a broad range of spectral wavelengths simultaneously containing factors related to analyte concentrations are considered. Interfering absorption bands, scattering differences and shifts in band positions are compensated for the definition of reliable calibration models.

MLR methods are less sensitive to subtle matrix variations [27]. In turn, PLS or PCR methods are usually employed for the analysis of more complex chemical systems (*i.e.*, quantification of minor constituents or when bands are highly overlapped).

The usefulness of a given model fully depends on the set of data used to create it, and on the goal of the analysis. The most critical aspect to be considered for the definition of calibration models is the ensuring that they will be really representative of variations encountered in future samples [28]. Therefore, it is advisable to use independent sets of samples collected from at least one independent experiment to validate the performance of calibration models.

## 4. Applications

### 4.1. Use of Vibrational Spectroscopic Methods in Taxonomy of Lactic Acid Bacteria and Probiotics

Even when the first approach to use infrared and Raman spectroscopy to analyze microorganisms dates back from 1951 [29], it was only in 1991, with the development of modern equipment and analytical methods, when the use of Fourier Transform Infrared Spectroscopy (FTIR) and Raman for taxonomic purposes acquired great interest among microbiologists [30].

FTIR and Raman spectra represent “signatures” of the overall bacterial chemical composition. Considering that chemical structures (*i.e.*, protein profile, fatty acid composition, polysaccharides, nucleic acids, *etc*.) have common patterns for genera, species, subspecies [31], vibrational spectra provide highly specific whole-microorganism phenotypic fingerprints, which is doubtless valuable for taxonomic purposes [30].

The development of sophisticated equipment has strongly contributed for registering high quality spectra of large amounts of samples in a short time. This was determinant to sustain its use as a reliable and rapid approach for the identification and classification of microorganisms at a species and even at the strain level [32]. This latter capacity enables the use of these techniques in epidemiological studies and also in certain technological applications, namely the analysis of starters and probiotic cultures [32,33,34,35,36,37,38].

Experimental design requires cultivation of microorganisms either in liquid or in solid medium. Liquid cultures are harvested by centrifugation and after removing the liquid supernatants, an aliquot of the obtained material is used for spectra registration. When grown in solid media, microorganisms are harvested with a loop and suspended in a small amount of water. In both cases, about 30 μL of microorganisms´ suspensions are placed on FTIR windows (in general, of zinc selenite, calcium fluoride or other water-insoluble and IR transparent material), spread to cover the whole pre-defined sample area and dried either in a desiccator over a drying agent (silicagel, P_4_O_10_) or under moderate temperature (40 °C) to avoid water interferences. The FTIR windows are then sealed in a gas-tight cuvette-cartridge to control humidity and to prevent contamination. All protocols, including cultivation conditions, harvesting, drying and registration of spectra, shall be strictly standardized to guaranty reproducibility of results [25].

Besides these considerations, it shall not been forgotten that the enormous diversity of microbial species and strains, requires a conscious use of multivariate methods for the analysis of data. The most widely used for microbial taxonomy include HCA, FA, and ANN [25].

Based on the similarities and differences among spectra, HCA enables creating dendrograms, that is, graphical plots grouping similar spectra. HCA is a suitable method when only a limited number of species are expected to be detected. However, to avoid misclassifications, it shall be used with caution and only by people having strong microbiological knowledge.

For identification purposes, and especially when largely different genera and species are to be investigated, the availability of broad and comprehensive databases is crucial to deal with the huge intraspecies diversity. These databases shall include different reference strains, desirably from different origin. This enables covering intraspecies diversity and ensures a reliable identification. To this purpose, other methods like ANN are more appropriate and have actually been used for the identification and differentiation of probiotics at the strain level [39,40]. Several authors contributed for the creation of spectral libraries of different species of the genera *Lacbobacillus*, *Lactococcus*, *Leuconostoc*, *Streptococcus*, *Pediococcus*, *Propionibacterium* and *Enterococcus* [32,40,41,42,43,44,45,46,47]. FTIR based techniques enabled the accurate identification of different lactic acid bacteria and probiotics isolated from rumen extracts [48], human feces [49] and food matrices like breweries [50], cheese and dairy products [51,52,53,54,55,56,57,58], water kefir [59], pickled and fermented vegetables food and juice, and salami [44,47], meat [60].

Prabhakar *et al.* (2011) [61] developed a classification method of *S. thermophilus*, *Propionibacterium freudenreichii* and *Lactobacillus* sp. at the strain level by combining hydrophobic grid membrane filters and FTIR. Collected spectra were statistically analyzed by SIMCA for pattern recognition. The classification models showed major discrimination in the 1200–900 cm^−1^, supporting their use as a potentially effective tool to identify and monitor the activity of cheese and other dairy starters.

*L. sakei* and *L. curvatus*, two closely related species occurring in meat could also be identified using FTIR and PLSR, even in large data sets [60]. More recently, Mouwen *et al.* (2011) [62] discriminated 55 strains (including 16 reference strains) of lactic acid bacteria from the genera *Lactobacillus* (species *sakei*, *plantarum*, *curvatus*, *brevis*, *farciminis* and *alimentarius*), *Weissella* (species *viridescens* and *halotolerans*) and *Carnobacterium* (species *maltaromaticum* and *divergens*) using FTIR, HCA and stepwise discriminant analysis (SDA), which were subsequently carried out to detect classes and create library groups.

Bosch *et al.* (2006) [34] differentiated 42 lactobacilli strains isolated from kefir grains by HCA, including 12 reference strains in the analysis. As a first step, they discriminated homo and heterofermentative strains and then, identified microorganisms of each group at the species level. The species *L. plantarum*, *L. acidophilus*, *L. kefirgranum*, *L. kefiranofaciens* and *L. casei* were clearly placed in the first group, and the species *L. kefir*, *parakefir* and *brevis*, in the second [34]. Microorganisms from this latter group could also be differentiated and classified using Raman spectroscopy in combination with PCA and PLS-DA [63].

Hammons *et al.* (2010) [64] found an interesting relation between the species of *Lactobacillus* (*agilis* and *johnsonii*) isolated from corn-soy broiler chicken diet and those present in the gut. They found that the nutritional environment in the chicken’s digestive tract select for *L. agilis* isolates with a specific ecophysiology. Using FTIR, they successfully characterized isolates of the species *L. agilis* and *L. johnsonii* directly on freshly prepared films of bacteria. Comparison of the second derivative spectra in the amide I and II regions resulted in an almost perfect grouping of the *L. agilis* isolates by diet [64].

Georges *et al.* (2008) [54] analyzed a cheese ripening consortium considering the question whether or not the inoculated commercial ripening starters are able to develop over the time of ripening and compete against the resident microbial flora. Samelis *et al.* (2011) [55] identified lactic acid bacteria flora present in traditional Greek Graviera cheese after five weeks of ripening and Lefier *et al.* (2000) [56] followed the evolution of *Lactococcus* strains during ripening of Brie cheese. In turn, Vodnar *et al.* (2010) [65] followed the lactic acid production of mixtures and pure strains of *L. plantarum*, *L. casei*, *B. infantis* and *B. brevis* during growth using HPLC, and obtained the microorganisms pattern using FTIR.

FTIR was also used with success to differentiate pathogenic from non-pathogenic strains of *Bacillus cereus*, known as probiotics, namely *Bacillus cereus* var. *toyoi*, which is used as Toyocerin^®^ powder (a product of heat-stable spores) in animal feed. Furthermore, *Bacillus cereus* CIP 5832 was used for the same purpose as the probiotic feed additive Paciflor^®^ and was retrieved from the global market in 2001 [66]. It was possible to separate probiotic *B. cereus* strains from wild-type *B. cereus*, *B. thuringiensis*, *B. mycoides*, *B. weihenstephanensis* using HCA. The main differences were observed in the fatty acid composition (3000–2800 cm^−1^ region), carbohydrates (1200–900 cm^−1^ region) and fingerprint region (900–700 cm^−1^) [67].

### 4.2. Use of Vibrational Spectroscopic Methods in the Preservation and Storage of Lactic Acid Bacteria and Probiotics

The industrial use of lactic acid bacteria requires the optimization of preservation and storage conditions. Freezing, freeze-drying and spray-drying are the most widely used preservation methods. All these methods lead to a decrease of water activity to slow down or avoid deteriorative reactions. This dehydration may conduct to the loss of structural water with a concomitant damage of membranes and proteins that finally results in the loss of bacteria viability. In general, sugars (*i.e*., trehalose, sucrose, maltose among others) and aminoacids are used to prevent these damages. Their protectant capacity has been explained on the basis of two hypotheses: (a) the water replacement, that is, the capacity of a given protectant to take the place of water and form hydrogen bonds with lipid membranes and/or other macromolecules; and (b) the formation of glassy states, that is, the formation of amorphous states where biochemical reactions are inhibited [68].

As formation and broken of hydrogen bondings is involved in both hypotheses, FTIR is very sensitive to evaluate the protectant effect of sugars and aminoacids. In fact, it has been widely used to investigate the ability of protective compounds to interact with polar heads of lipid membranes in both model systems and intact cells, including lactic acid bacteria [25,69,70,71,72]. The hydroxyl groups of protectants (in general sugars or polyols) interact with phosphates through hydrogen bondings. In the absence of water (when no protectant is used), one can observe noticeable shifts of the phosphate asymmetric stretching vibrational mode (~1230 cm^−1^) to higher wavenumbers [73]. On the contrary, in the presence of sugars, this band occurs at the same wavenumber as that observed in hydrated membranes [69,73].

Molecules of higher molecular weight, like maltodextrines or hydroxyethyl starch, are not able to replace water in lipid membranes. However, their presence facilitates the interaction of smaller sugars (*i.e.*, sucrose, glucose) with lipids [74,75].

The fatty acid composition of lipid membranes (namely, the saturated/unsaturated ratio and the chain length) determines the melting temperature (Tm), that is, the temperature at which the membrane pass from the gel to the liquid crystalline state. The dihedral angles established by the carbon atoms of the hydrocarbon chains are in *trans* conformation in the gel state, and in *gauche* conformation in the liquid crystalline phase. These two conformations lead to noticeable differences in the position of both the symmetric and asymmetric CH_2_ stretching vibrational modes (2850 and 2920 cm^−1^, respectively), thus supporting the use of FTIR spectroscopy to determine the Tm of liposomes and intact cells [76].

The loss of hydration water from polar head groups occurring during preservation processes leads to changes in the packing of fatty acids, which in turn may alter the membrane permeability, conducting to leakage of the bacterial intracellular content. When dehydrating in the absence of sugars, membrane lipids become highly packed and therefore, Tm increases. When sugars are present during dehydration, they interact with phosphate groups and Tm remains close to that of hydrated membranes. This explains their role in preserving membrane properties and in increasing the survival of dehydrated probiotics [69,77]. Indeed, the protective effect of trehalose, sucrose and sorbitol on lipid membranes has been reported for several species of lactic acid bacteria, including *Lactococcus lactis* [76], *Bacillus thuringiensis* [77], *L. plantarum* [69], *L. helveticus* [72] and *L. delbrueckii* subsp. *bulgaricus* [74]. In turn, alterations in the fatty acid composition of *L. bulgaricus* are directly related with the composition of the culture medium. Growing microorganisms in a rich culture medium like MRS, is associated with higher contents of cyc 19:0 fatty acid, in comparison with microorganisms grown in a whey-based medium (less rich medium). This higher content of cyc 19:0 fatty acid is related with the lower Tm and higher resistant of microorganisms grown in MRS medium to freezing, as shown by FTIR [78].

Proteins are other targets of damage during bacteria preservation. Changes in the amide bands are useful to evaluate denaturation, or aggregation of intracellular proteins by FTIR [76]. There are four main amide bands: amide A (3400 cm^−1^) [76], amide I (1655 cm^−1^), amide II (1550 cm^−1^) and amide III (1250 cm^−1^) [78]. Amide A occurs at about 3500 cm^−1^ and arise from the N-H vibrational mode. It does not depend on the skeletal conformation, but is very sensitive to the strength of hydrogen bonds. Amide I arises from the carbonyl stretching vibration of the peptide bonds. It is the most useful band for the study of the secondary structure of proteins and peptides. Amide II and Amide III originate in the coupling of the N-H bending and C-N stretching vibrational modes [79]. Sugars can also interact with dehydrated proteins maintaining their folding, and this capacity can be monitored by analyzing the proteins’ amide bands [74]. Killiman *et al.* (2006) [76] reported increase in thermal resistance of *Lactococcus lactis* in the presence of sucrose by analysis of amide bands in deuterium oxide (D_2_O). The H/D exchange in proteins provides information about the stability and flexibility of proteins, structural hydrogen bonds being more protective less prone to be interchanged.

The second hypothesis explaining the protective capacity of sugars is related with their capacity to form glassy (or amorphous) states, in which the high viscosity and low mobility restrict molecular interactions [80,81,82,83]. Glass transitions occur at a given temperature and are dependent on the water content. Therefore, samples shall remain in amorphous state (below T_g_) for a successful storage [84,85].

The amorphous state is characterized by weak hydrogen bondings between sugar and water. On the contrary, in the rubbery state (that is, above Tg), the interaction between water molecules leads to stronger hydrogen bondings. The change in the hydrogen bonding networks (*i.e.*, length and strength) is reflected in the FTIR spectra, which supports using FTIR to determine Tg [71,74,86]. When going from the amorphous to the rubbery state, a dramatic shift of the OH stretching vibrational mode to lower wavenumbers is observed [74,87]. Other vibrational modes, like the bending OH (~1030 cm^−1^) and the first overtone of the symmetric valence vibration (~6800 cm^−1^) also provide information [10,74,88].

FTIR also enables the determination of structural alterations resulting from the exposure of lactic acid bacteria to adverse environments including non-favorable growth conditions [89,90] and dehydration by vacuum drying. In this latter case, the main differences are observed in the bending of CH_2_ and CH_3_ (1400–1450 cm^−1^) and in the fingerprint region of carbohydrates (1200–900 cm^−1^) [91].

### 4.3. Use of Vibrational Spectroscopic Methods in Monitoring Lactic Acid Bacteria and Probiotics

Vibrational spectroscopic methods are particularly useful at an industrial level and in applied sciences because of their feasibility for monitoring biological processes in real time, without the necessity of exogenous chemical reagents. Furthermore, the simplicity of interfacing the modern spectroscopic equipment to almost any computer or dataset converts these methods in useful tools for automatic control, process optimization or quality assessment. This supports the increasing interest of both enterprises and academia in using them for the monitoring of different industrial processes, including fermentations, quality control, biomass production among others.

NIR is the most used approach to follow fermentations. The NIR spectra can be registered: (a) *Off-line* (spectra are collected at a given time and analyzed later, generally in a different place); (b) *at-line* or rapid *off-line* (spectra are collected and analyzed immediately after being collected); (c) *on-line* (spectra are collected and analyzed at the moment without manual sample manipulation). In turn, two types of *on-line* monitoring can be considered: *in situ* or *in line* and *ex situ.* The first type of monitoring is useful in the production line, as spectral information is transported from the sample to the spectrometer through a fiber-optic probe immersed into the fermentation broth [92,93]. For *ex situ* monitoring, the analysis device is physically outside the fermenter and spectra are usually registered with either a flow-through cell or a fiber optic probe placed on the glass wall of the container.

One of the first articles dealing with the monitoring of lactic acid fermentation was published by Vaccari *et al.* (1994) [94]. They used reflectance techniques of NIR for the *on-line* determination of *L. casei* in continuous fermentation processes. Later on, Gonzalez-Vara *et al.* (2000) [95] designed a NIR-based control system to full automate the production of lactic acid by *L. casei* at a small-scale pilot plant. Besides lactic acid, the system also provided information on biomass and glucose. Vodnar *et al.* (2012) [96] obtained qualitative and semi-quantitative information for lactic acid production by growing *L. casei* in MRS and in MRS supplemented with high concentrations of fructans (dandelion extract).

The concentrations of lactose, galactose, lactic acid and biomass in fermentation processes were determined using combined approaches of FTIR and PLS, and HPLC as reference method [97,98]. In a later work, Fayolle *et al.* (2000) [99] developed a system consisting in a FTIR spectrometer coupled with an attenuated total reflectance probe by optical fibers to *on-line* analyze both substrates (glucose, fructose, lactose and galactose) and products (ethanol and lactic acid) of alcoholic and lactic fermentations. In turn, Sivakesava *et al.* (2001) [100] used mid and near infrared, and Raman spectroscopies for *at-line* monitoring the production of biomass, glucose and lactic acid by *L. casei*. Suitable spectral wavenumber regions were selected to calibrate PLS and PCR models.

Macedo *et al.* (2002) [101] defined a PLS model to determine the concentrations of exopolysaccharide, lactic acid and lactose in the supernatants of *L. rhamnosus* RW9595M cultures by using second derivative NIR spectra and Grassi *et al.* (2013 and 2014) [102,103] monitored the fermentation of single or mixed cultures of *S. thermophilus* and *L. delbrueckii* subsp. *bulgaricus* to obtain information about quality parameters, including bacterial counts, titratable acidity, pH, lactose, galactose and lactic acid concentrations. Oberreuter *et al.* (2000) [104] also determined the ratios of different food associated species in binary mixtures, but the mixtures analyzed included *Saccharomyces cerevisiae*/*Hanseniaspora* *uvarum* and *L. acidophilus*/*S. thermophilus*.

Nicolau *et al.* (2011) [105] detected and enumerated *Staphylococcus aureus* in milk, as well as the growth interaction between *S. aureus* and *Lactococcus lactis* ssp. *cremoris*. Both species were investigated as pure cultures and in co-cultures after inoculation into UHT milk. Definition of PLS based models using plate counting as reference method, enabled an accurate and sensitive detection of the mentioned microorganisms directly from the FTIR and Raman spectra [105].

Cimander *et al.* (2002) [106] followed yogurt fermentation at a laboratory scale using a combination of electronic nose, NIR and standard bioreactor probes. Using a cascade neural network, they fused the sensor signals as follows: a primary network to predict quantitative process variables (*i.e.*, lactose, galactose, and lactate), and a secondary network to predict a qualitative process state variable describing critical process phases (*i.e.*, onset of coagulation, harvest time). This approach improves the quality control of yoghurt fermentation by simultaneously predicting both quantitative and qualitative process’ variables. Navraätil *et al.* (2004) [107] used NIR and electronic noses for *on-line* monitoring fermentations leading to the production of yogurt and fimjölk, a Swedish yogurt-like sour milk, under industrial conditions. First, PCA was carried out and then, the first PCs were used for *on-line* generation of a plot indicating the evolution of the fermentation process. With that background, an acceptable PLS model was defined to predict pH and titratable acidity [107].

Da Cruz *et al.* (2009) [108] developed an approach based on ANN to monitor the authenticity of commercial yogurts. To this aim, they built a data base using 108 strawberry-flavored yogurts (48 probiotic low-fat, 36 low-fat, and 24 full-fat yogurts) belonging to several commercial brands and from different batches. The model was successfully validated with independent data pairs. Rodriguez *et al.* (2013) [109] used Raman spectroscopy to follow kinetics of fermentation in yogurt elaboration.

Santos *et al.* (2014) [110] monitored the kinetics of growth of *L. delbrueckii* subsp. *bulgaricus* after dehydrating microorganisms in the presence of galactooligosaccharides and lactulose. The relative composition of microorganisms (viable, damaged or dead) throughout the kinetics of growth was assessed by multiparametric flow cytometry, using carboxyfluorescein diacetate and propidium iodide probes. PCA performed on the second derivative NIR spectra showed three groups along PC1, corresponding to the *lag*, exponential and stationary phases of growth, which explained 99% of the total variance. Along PC2, two groups were observed, corresponding to damaged and viable cells [110].

Sorghum is generally fermented with commercial strains of *L. plantarum*, *L. brevis*, *L. paracasei*, *L. fermentum*, *Pediococcus pentosaceus* and *S. thermophilus*. As a result of fermentation, sorghum prolamins are hydrolyzed and *in vitro* digestibility is facilitated. The fermentation process can be followed by FTIR. In a first paper, Correia *et al.* (2005) [111] inoculated wet-cooked sorghum flour with *L. fermentum*, *L. bulgaricus*, *L. lactis*, *Pediococcus pentosaceus* and *P. cerevisiae* and also with a mixture of *L. bulgaricus* and *S. thermophilus* from a commercial yogurt. The increase of titratable acidity, total protein content and free aminoacids, and the decrease of reducing sugars, soluble proteins and starch during fermentation were analyzed using traditional chemical methods and confirmed by ^1^H NMR and FTIR, thus supporting their use to follow these fermentation processes [111]. In a later work, the same group of authors correlated the structural differences of the fermented starches (as determined by FTIR) with the species of lactic acid bacteria inoculated in sorghum [112].

Johnson *et al.* (2004) [113] investigated the dynamics of red clover or grass silage fermentations in response to various types of starter inoculants associating FTIR with PCA that enabled the discrimination between herbage types and different lactic acid bacteria inoculants. Amide I and amide II regions were those where the greatest differences among treatments were observed.

Aliakbarian *et al.* (2015) [114] used NIR to characterize the product obtained by adding aqueous phenolic extracts of olive and grape pomace, to skim milk (fermentation medium). NIR associated with PCA and LDA enabled the discrimination of samples according to the type of extract added.

Infrared spectroscopy has been widely applied in winemaking to quick perform a quality control of the product at all stages of the process. This analysis includes assessing both substrate and product concentrations (*i.e.*, sugars, ethanol) and also other quality parameters such as phenolic composition and volatile compounds. All these parameters can be followed during wine production by using FTIR and chemometric analysis [115,116].

Other interesting applications of FTIR have been used in Asia. Rice fermentation is an important step in the elaboration of rice paper because it changes physicochemical and morphological properties of rice flour and also influences the final product. This fermentation step results from the activity of different genera of lactic acid bacteria (*Lactobacillus*, *Lactococcus*, *Leuconostoc*, *Pediococcus*) and leads to a decrease of moisture, starch and lipid content and an increase of protein conntrations. Therefore, FTIR analysis can be used to investigate the changes experienced by rice during fermentation [117].

Certain species of lactobacilli are associated with meat spoilage. Different authors used NIR to detect them. Argyri *et al.* (2010) [118] correlated meat spoilage during aerobic storage at 0, 5, 10, 15 and 20 °C with the FTIR spectra. The FTIR were collected directly from the surface of meat. On the basis of the biochemical information provided by the FTIR spectra an ANN was designed to classify beef samples. These results were correlated with the viable plate counts. The network was able to classify correctly 22 out of 24 fresh samples (91.7%), 32 out of 34 spoiled samples (94.1%), and 13 out of 16 semi-fresh samples (81.2%). Results demonstrated good correlation of microbial load on beef surface with spectral data [118]. In a later study, the same group of research demonstrated that Raman spectroscopy is as well as FTIR in predicting meat spoilage [119]. Similar approaches were followed by other authors, who used supervised chemometric methods to count bacteria and also to quantify biochemical changes occurring during storage [120,121,122,123].

Camara-Martos *et al.* (2011) [124] used NIR to identify and quantify bacteria species in water-based systems, simulating water-based food matrices. To this aim, they investigated three species associated with spoilage in ready-to-eat meat (*i.e.*, *L. plantarum*, *Leuconostoc mesenteroides* and *L. sakei*) in the 1100–2500 nm region. PCA enabled a clear discrimination among the three species and PLS enabled a successful quantification in the range 3–9 log CFU/mL, and also the possibility of distinguishing between spoilage (7–9 log cfu/mL) and non-spoilage concentration levels (3–6 log cfu/mL) [124].

McKinney *et al.* (2012) [125] used FTIR and Raman spectroscopies to assess the mechanisms involved in the protective capacity of *L. crispatus* to inhibit the pathogenic foodborne bacteria *Camylobacter jejuni*. The harmful effect of *L. crispatus* is given by lactic acid. Spectral features of *C. jejuni* and *L. crispatus* enabled the accurate prediction of bacterial viability and also, the discrimination of *C. jejuni* according to the treatment received. When both microorganisms are grown in co-culture, the metabolism was dominated by *Lactobacillus* prior to the killing of *C. jejuni* [125].

Other authors demonstrated the reliability of vibrational spectroscopies to determine the capacity of lactic acid bacteria to remove harmful concentrations of heavy metals [126].

### 4.4. Use of Vibrational Spectroscopic Imaging Techniques in the Study of Lactic Acid Bacteria and Probiotics

Hyperspectral chemical imaging is a broad term referring spatially resolved spectral information obtained through different techniques, namely Raman, FTIR, NIR and fluorescence. Using these techniques for investigation of lactic acid bacteria and probiotics is very scarce and most of publications are focused on the effects of microorganisms on eukaryotic cells or tissues rather than on bacteria themselves. For example, Giorgini *et al.* (2010) [127] reported the structural changes observed in oocytes after treatment with *L. rhamnosus* IMC 501 for 10 days. He *et al.* (2014) [7] reported NIR images of the salmon surface obtained as result of the spoilage effect of lactic acid bacteria, and Dissing *et al.* (2013) [128] quantified the degree of spoilage of stored minced pork meat. Daniel *et al.* (2014) [129] observed structural changes in mice gut microbiota physiology resulting from differences in diet. Other example is the use of Raman microspectroscopy together with FISH for physiological characterization and identification of individual cells in the complex intestinal microbiota [130].

## 5. Conclusions

Vibrational spectroscopic techniques were born as almost exclusive physical and chemical tools generally employed to resolve the chemical structure of compounds having a reduced number of atoms. The development of strong equipment and computational resources certainly contributed to the growth of novel research areas at the interface between Biosciences and Physical-Chemistry. The enormous advantage of these techniques is that they do not require external reagents (they are environmentally friendly) and sample preparation is minimal, which is particularly relevant for industrial applications. However, the inherent complexity of biological samples requires a strong background on mathematical and statistical tools (Chemometrics) for spectral interpretation. Unfortunately, sometimes this is not a common skill among researchers with biological background.

In this review, we put into relevance the use and potentialities of this approach in studying lactic acid bacteria and probiotics both at an industrial and at a research level. Considering the great importance of probiotics market, the systematic industrial implementation of these techniques could be much more explored than it is. In regard to research, it is our opinion that using vibrational spectroscopic techniques to investigate lactic acid bacteria and probiotics represents a relatively novel approach far from being fully explored and where a lot of things still remain to be done.
